# Supplementary material for: AI specialization for pathways of economic diversification
Source: Sci Rep. 2023 Nov 9;13:19475. doi: 10.1038/s41598-023-45723-x (PMC10636127; doi:10.1038/s41598-023-45723-x)
Supplement: Supplementary file 1 — Supplementary Information. [file 41598_2023_45723_MOESM1_ESM.pdf]

AI Specialization for Pathways of Economic Diversification
-
Supplementary Information

Saurabh Mishra[1], Robert Koopman[2], Giuditta De-Prato[3], Anand Rao[4], Israel Osorio-Rodarte[5], Julie Kim[6], Nikola Spatafora[7], Keith Strier[8], and Andrea Zaccaria[9]

[1]Institute for Human-Centered Artificial Intelligence (HAI), Stanford University, [2]World Trade Organization (WTO), [3]Joint Research Center (JRC), European Commission, [4]PricewaterhouseCoopers (PwC), [5]World Bank Group, [6]Netbase Quid, [7]International Monetary Fund (IMF), [8]NVIDIA Corp., [9]Institute for Complex Systems, CNR

September 29, 2023

Data Description

AI Investment

Organization data is embedded from CapIQ and Crunchbase. These companies include all types of companies (private, public, operating, operating as subsidiary, out of business) in the world; The investment data include private investment, Mergers & Acquisitions (M&A), public offerings, minority stakes made by Private Equity/Venture Capital firms, corporate venture arms, governments, and institutions across more than 80 countries. Some data is simply unreachable when the investors are undisclosed, or the funding amounts by investors are undisclosed. We also embed firmographic information such as the year founded and HQ location. Quid embeds CapIQ data as a default and we add data from Crunchbase for the ones that are not captured in CapIQ. This way we not only have comprehensive and accurate data on all global organizations but also capture early-stage startups and funding events data that are publicly available.

A boolean search for "artificial intelligence" OR "AI" OR "machine learning" OR "deep learning" yields our source of private investments in AI. Private

investment is a private sale of newly issued securities (equity or debt) by a company to a selected investor or a selected group of investors. The stakes that buyers take in private placements are often minority stakes (under 50%), although it is possible to take control of a company through a private placement as well, in which case the private placement would be a majority stake investment. As AI investment is a relatively new field, our data is focused on the time period between 2010 and 2020. The complete list of AI sectors and sub-sectors is reported in Table S.1.

Service Exports

The source of the service exports analysis relies on Balance of Payments Manual (BPM6) classification of disaggregated data on credit accounts of services at 1-digit level. The BPM6 provides credit, debit and net accounts for services between 2010-2020. The coverage of trade in services data is still very limited and often unbalanced. Services, in contrast to goods, are characterized by several features, such as intangibility and non-storability, which complicate the collection of accurate international trade in services statistics [1]. In the spirit of [2, 3, 4] the combined goods and services dataset of world trade has been referred to as the universal matrix of world trade.

Goods Exports

Merchandise trade data was obtained from the U.N. Comtrade database available through the World Bank World Integrated Trade Solution System (WITS[1]). The Standard International Trade Classification (SITC) Revision 2 is preferred, which contains 786 items at its 4-digit level. The SITC Revision 2 was published in 1975 and the majority of countries continue to use it for various purposes, such as study of long-term trends in international merchandise trade and aggregation of traded commodities into classes more suitable for economic analysis. More recent revisions, nonetheless, have new set of product categories that have been created in recent years.[2] Since countries report both their imports and their exports to the United Nations, the raw data duplicates flows . As a result, there is a process for reconciling data due to well-known discrepancies in trade statistics. In general terms, we consider mirror statistics for fragile countries low-income countries.

Using the trade data, standard metrics of trade were constructed following [5] a series of network metrics were calculation on top of the merchandise trade data, such as revealed comparative advantage, proximity, and density. In addition, the product space shows that products group naturally into highly connected communities. This suggest that product in these communities are closely connected

[1]The World Integrated Trade Solution (WITS) software provides access to international merchandise trade, tariff and non-tariff measures (NTM) data. https://wits.worldbank.org

[2]The SITC classification reflects (a) the materials used in production, (b) the processing stage, (c) market practices and uses of the products, (d) the importance of the commodities in terms of world trade, and (e) technological changes. https://unstats.un.org/unsd/classifications/Family/Detail/1070

to each other than products outside of the community. Following [6] product communities complement the trade database. These communities were originally created using the two-step algorithm introduced by [7].

Tables and Figures

Table S.1: AI sectors used and their sub-sectors disaggregation

AI Investment sectors used	AI sub-sectors included
Advertising	Advertisers, Programmatic, Mobile advertising, Real time bidding, Influencer, Social media marketing, Content marketing, Advertisers
AgTech	Agriculture, Farmers, Farming, Crop
AR, VR	Augmented reality, Vr, Virtual reality, Ar
Authentication Technology	Facial, Face recognition, Law enforcement, Video surveillance
Autonomous Vehicle	Autonomous vehicles, Fleet, Road, Autonomous driving
Cancer, Drug Discovery	Drug, Cancer, Therapy, Genomic
Computing Technology	Quantum, Quantum computing technologies, Applications for quantum, Simulation of quantum, Semiconductor, Chips, Processors, Low power
Crypto, Wealth Management	Crypto, Wealth management, Traders, Cryptocurrency
Data Science, Data Platform	Data centers, Migration, Cloud management, Application performance, Reinforcement learning, General intelligence, Sift through data, Platform for AI, Sql, Hadoop, Python, Data preparation
Drone, Satellite	Drone, Satellite, Unmanned, Remote sensing
Education	Student, Edtech, Children, Career
Energy Management	Energy management, Buildings, Renewable, Electricity
Fitness and Wellness	Wellness, Wearable, Fitness, Emotions
Fraud Detection, Money Laundering	Fraud detection, Merchants, Laundering, Personal finance
Gaming, e-sports	Player, Esports, Mobile games, Fans
Hospitality	Hotels, Booking, Business travel, Online travel
Insuretech	Insurtech, Insurance industry, Underwriting, Insurance products
Legal Tech	Legal, Law, Contract management, Lawyers
Media Content	Editing, Instagram, Photo sharing, Reserve a table, Topics, Video content, Readers, Personalized content
Medical Technology	Doctors, Hospital, Physicians, Medication, Medical device, Surgical, Blood, Cardiac
Money, Personal Finance	Invoices, Medium businesses, Cash flow, Receipts, Lending, Loans, Credit score, Consumer finance
Network Security	Threat, Network security, Cybersecurity, Security solutions
Oil and Gas	Gas, Predictive maintenance, Industrial automation, Machinery
Real Estate	Commercial real estate, Landlords, Estate agents, Property management
Recruiting	Recruiting, Candidate, Hiring process, Recruiters
Restaurants, Food and Beverage	Food and beverage, Kitchen, Grocery, Food delivery
Retail Tech, E-Commerce	Ecommerce, Marketing automation, Shoppers, Retail technology
Robotic Automation	Industrial automation, Ai robotics, Mobile robot, Warehouse management, Robotic process automation, Rpa, Test automation, Business process automation
Sales Automation, Meeting Management	Meetings, Sales automation, Prospects, Sales teams

Supply Chain Management	Supply chain management, Freight, Shipping, Procurement process
Text Analytics	Bots, Chatbots, Conversational ai, Messenger, Sentiment, Customer feedback, Employee experience, Text analytics, Speech recognition, Musical, Podcasts, Songs
Venture Capital	Equity, Technology startups, Mentorship, Platform for startups
Wifi, Home Tech	Requires ios, Ipod touch, Requires ios compatible, Compatible with iphone, Wi fi, Indoor, Wifi, Lights
Image Recognition, Visual Search	Fashion, Visual search, Shoes, Apparel
Neuroscience	Palo alto, Semantic analysis technology, Knowledge mapping, Public opinion monitoring

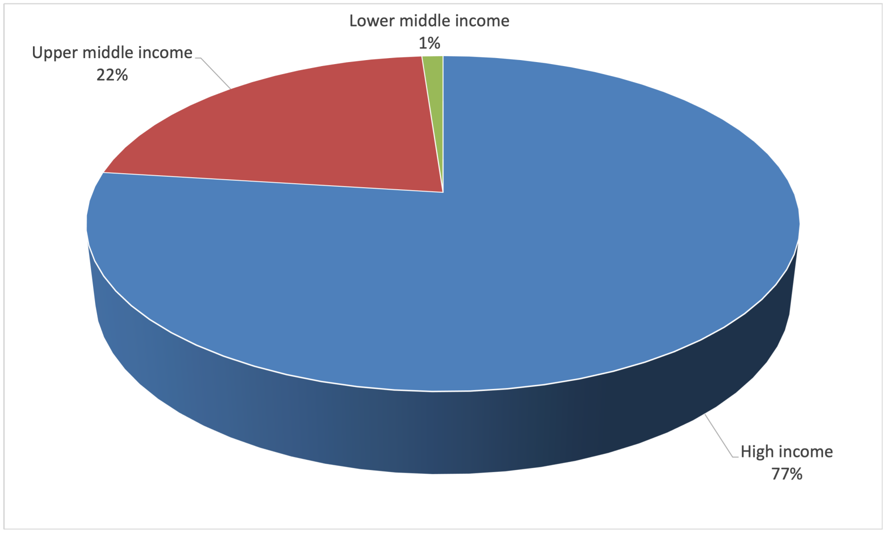

Figure S.1: Percent of global AI private investment by World Bank income groups, 2019-2020.

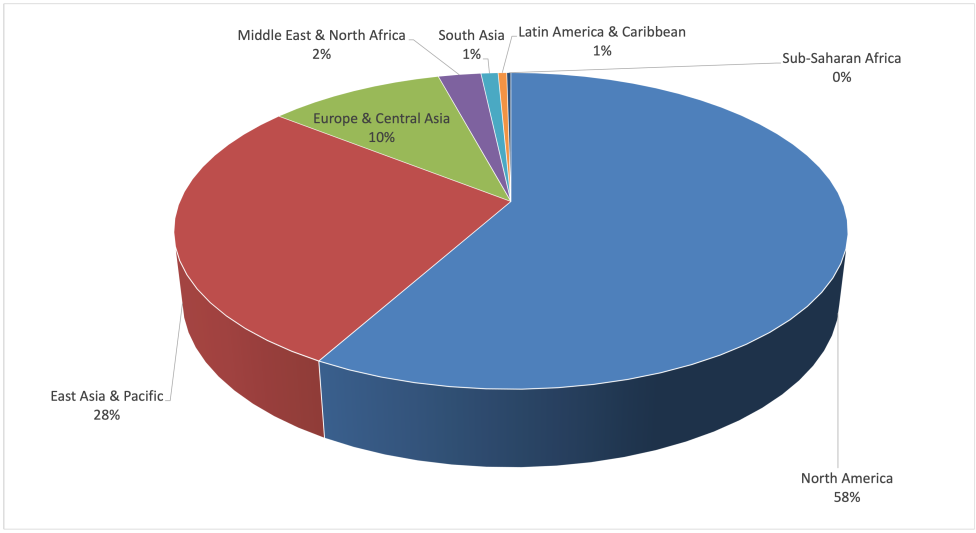

Figure S.2: Percent of global AI private investment by World Bank regions, 2019-2020.

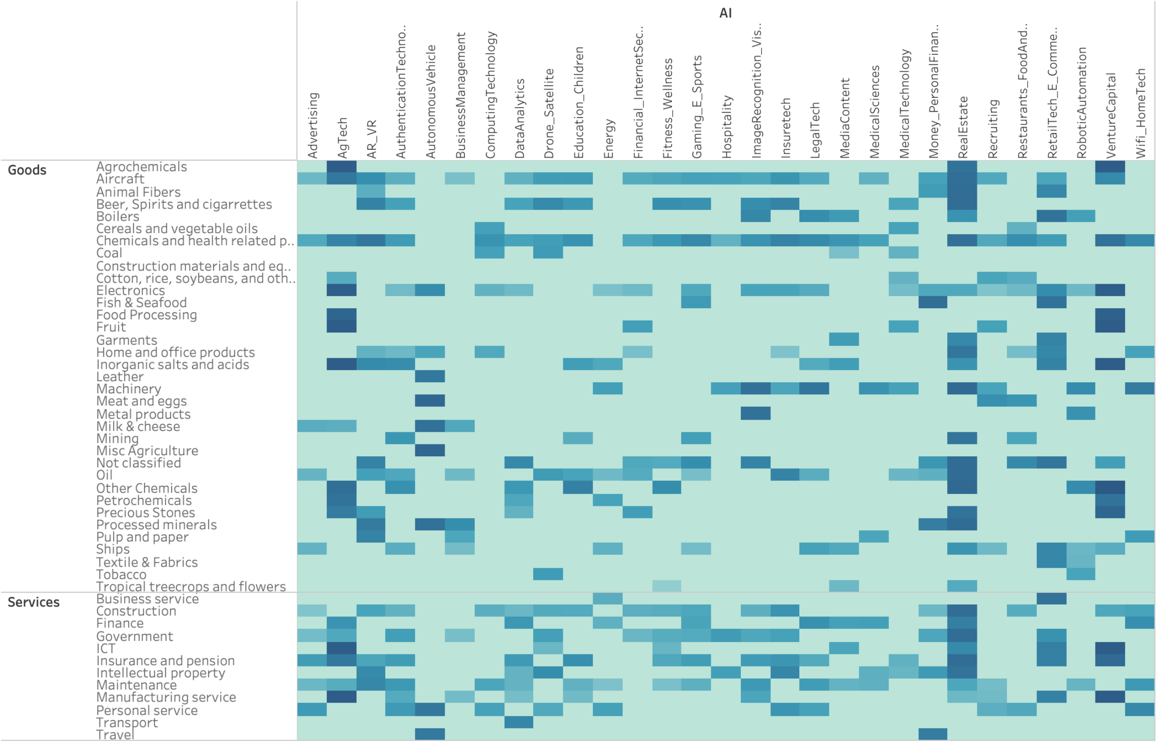

Figure S.3: Heatmap presentation of linkages from AI investment to goods and services export specialization. The darker shade of blue indicates statistically validated stronger linkages.

References

[1] F Spinelli and S Miroudot. Estimating bilateral trade in services by industry–the ebtsi dataset, 2015.

[2] Viktor Stojkoski, Zoran Utkovski, and Ljupco Kocarev. The impact of services on economic complexity: Service sophistication as route for economic growth. *PloS one*, 11(8):e0161633, 2016.

[3] Andrea Zaccaria, Saurabh Mishra, Masud Z Cader, and Luciano Pietronero. Integrating services in the economic fitness approach, 2018.

[4] Saurabh Mishra, Ishani Tewari, and Siavash Toosi. Economic complexity and the globalization of services. *Structural Change and Economic Dynamics*, 53:267–280, 2020.

[5] Ricardo Hausmann and Bailey Klinger. Structural transformation and patterns of comparative advantage in the product space, 2006.

[6] Ricardo Hausmann, César A Hidalgo, Sebastián Bustos, Michele Coscia, and Alexander Simoes. *The atlas of economic complexity: Mapping paths to prosperity.* Mit Press, 2014.

[7] Martin Rosvall and Carl T Bergstrom. Maps of random walks on complex networks reveal community structure. *Proceedings of the National Academy of Sciences*, 105(4):1118–1123, 2008.
